# Plant species within Streptanthoid Complex associate with distinct microbial communities that shift to be more similar under drought

**DOI:** 10.1002/ece3.11174

**Published:** 2024-03-24

**Authors:** Alexandria N. Igwe, Ian S. Pearse, Jessica M. Aguilar, Sharon Y. Strauss, Rachel L. Vannette

**Affiliations:** ^1^ Entomology and Nematology University of California – Davis Davis California USA; ^2^ Department of Biological Sciences Virginia Tech Blacksburg Virginia USA; ^3^ Evolution and Ecology University of California – Davis Davis California USA

**Keywords:** drought, plant–microbe interaction, rhizoplane, serpentine, streptanthus

## Abstract

Prolonged water stress can shift rhizoplane microbial communities, yet whether plant phylogenetic relatedness or drought tolerance predicts microbial responses is poorly understood. To explore this question, eight members of the *Streptanthus* clade with varying affinity to serpentine soil were subjected to three watering regimes. Rhizoplane bacterial communities were characterized using 16S rRNA gene amplicon sequencing and we compared the impact of watering treatment, soil affinity, and plant species identity on bacterial alpha and diversity. We determined which taxa were enriched among drought treatments using DESeq2 and identified features of soil affinity using random forest analysis. We show that water stress has a greater impact on microbial community structure than soil affinity or plant identity, even within a genus. Drought reduced alpha diversity overall, but plant species did not strongly differentiate alpha diversity. Watering altered the relative abundance of bacterial genera within Proteobacteria, Firmicutes, Bacteroidetes, Planctomycetes, and Acidobacteria, which responded similarly in the rhizoplane of most plant species. In addition, bacterial communities were more similar when plants received less water. *Pseudarthrobacter* was identified as a feature of affinity to serpentine soil while *Bradyrhizobium*, *Chitinophaga*, *Rhodanobacter*, and *Paenibacillus* were features associated with affinity to nonserpentine soils among *Streptanthus*. The homogenizing effect of drought on microbial communities and the increasing prevalence of Gram‐negative bacteria across all plant species suggest that effects of water stress on root‐associated microbiome structure may be predictable among closely related plant species that inhabit very different soil environments. The functional implications of observed changes in microbiome composition remain to be studied.

## Graphical abstract

Prolonged water stress has a significant impact on rhizoplane microbial communities in various plant species within the *Streptanthus* clade. This study found that the influence of water stress on microbial community structure is more pronounced than the effect of soil affinity or plant species identity. Drought reduced overall microbial diversity and led to similar changes in the relative abundance of certain bacterial genera across different plant species.

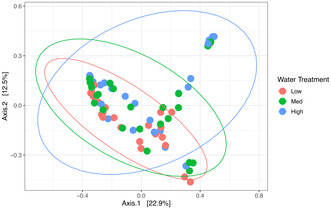

## 1 INTRODUCTION

Up to 70% of US land area has experienced abnormally dry periods from 2000 to 2020 (United States Environmental Protection Agency, 2021). Drought is expected to impact over 75% of the global population by 2050. Mitigating land degradation through soil and biodiversity conservation is one way to combat the impact of drought (United Nations, 2022). Many plant species have evolved physiological adaptations to withstand drought, including shorter heights, smaller leaves and fewer stomata (Brady et al., 2005; Grant, 2012; Heschel et al., 2017). In addition to, and sometimes in lieu of, those traits, microbial associations can alter plant phenotype and the environmental range of conditions plants are able to tolerate, including drought (David et al., 2019; Lau & Lennon, 2012). Plants can enrich the abundance of drought‐tolerant microbes or alter microbial composition in the rhizosphere which can protect plants from water stress by maintaining essential functions under stressful conditions (Xu & Coleman‐Derr, 2019). For example, due to their thick peptidoglycan layer, Gram‐positive bacteria are considered more drought tolerant than Gram‐negative bacteria and are often enriched under drought conditions (Chen et al., 2020; Chodak et al., 2015). In particular, many endospore‐forming Firmicutes and Actinobacteria have been shown to increase in abundance under water stress (Wang et al., 2020). Plant–microbe interactions can influence plant reproduction, distribution and biodiversity; therefore, understanding how plants and their rhizoplane microbial communities respond to and tolerate extreme climate events, such as drought, is increasingly important for the maintenance of plant diversity, production, and ecosystem functions across systems undergoing rapid changes in water regimes (Cook et al., 2018).

Serpentine soil is a naturally stressful soil environment with low essential plant nutrients and low‐water‐holding capacity, making it drought prone (Safford et al., 2005). These soil properties pre‐expose rhizosphere, and the more closely root‐associated rhizoplane microbial communities, to stressful conditions which may elevate the abundance of drought‐tolerant taxa compared to the rhizoplane microbial communities from plants grown in non‐serpentine soils. Under periods of water stress, drought‐tolerant microbial community members from serpentine soil may proliferate and protect the plant from future drought events (Bouskill et al., 2013; de Oliveira et al., 2020). Selection of drought‐resistant microbes through successive environmental filtering is one way that root‐associated microorganisms from drought‐prone soils may enhance plant tolerance to abiotic stress. Still, it is the interaction between abiotic conditions, plant selection, and source microbes that shape rhizoplane microbial communities.

Plant–microbe interactions in non‐managed systems may offer distinct insights into plant adaptations and responses to drought because plant species that can tolerate naturally drought‐prone soils may also be uniquely able to resist perturbation by drought by leveraging associations with soil microorganisms (Brady et al., 2005). In addition, closely related plant species can be strongly affected by underlying soil characteristics and still have distinct microbial communities when grown in a common soil environment (Burns et al., 2015; Erlandson et al., 2018). Furthermore, the dominant and rare taxa within a microbial community may have distinct influences (Xu et al., 2021). Experiments examining the relative contributions of soil type and species on structuring rhizoplane microbial communities as well as exploring the variation in ecology of rare and dominant taxa will yield insights into how rhizoplane microbial communities of closely related species may be impacted by the same stress event (Brady et al., 2005; Naylor et al., 2017).

Here, we assess the effect of drought stress on rhizoplane (a subset of the rhizosphere that is directly adjacent to the plant root) microbial community composition of eight wildflower plant species within the *Streptanthus* clade. Many members of this clade are endemic to California with varying drought tolerance and are a conservation priority (Kruckeberg & Morrison, 1983). We test the hypothesis that the rhizoplane microbial communities associated with *Streptanthus* species that have an affinity to serpentine soil will be less impacted by water stress. Using 16S rRNA amplicon sequencing we aimed to answer the following questions: (1) How does watering treatment and soil affinity impact the alpha diversity of bacterial phyla? (2) How does watering treatment shift community composition of rhizoplane bacteria and which bacteria are enriched under drought? (3) Does plant phylogeny or the watering regime of the natural habitat influence the response of rhizoplane microbial communities to water stress? (4) Do results differ if all bacterial taxa or only rare members are considered?

## 2 METHODS

### 2.1 Study system

The “Streptanthoid Complex” is a clade composed of several genera within the *Brassicaceae* family including *Streptanthus* spp. and *Caulanthus* spp. (Burrell et al., 2011; Pepper & Norwood, 2001). Most members of this complex are annual plants that occupy rocky outcrops and slopes (Al‐Shehbaz & Mayer, 2008). Their presence on these bare habitats are generally a precursor to endemism, which is relatively common within the clade (Armbruster, 2014; Cacho et al., 2014; Cacho & Strauss, 2014). This clade is composed of ~40 species with a range of edaphic specialization (i.e., serpentine tolerant and intolerant) (Burrell et al., 2011), making it an excellent system to study rhizoplane microbial community response to water stress. We chose eight species (populations within each species) within this clade, four with an affinity for serpentine soils and four without an affinity for serpentine soils (Table 1; The Calflora Database, 2016). Serpentine affinity varies among species in this clade, from serpentine specialists to species with populations that can grow across a large range of substrates, including serpentine.

**TABLE 1:** Species names, serpentine affinity, and natural rainfall.

Plant species	Plant species code	Soil affinity of population	Soil affinity of species as a whole	Rainfall (cm)
*Caulanthus amplexicaulis* var *amplex*	AMA	Nonserpentine	Serpentine‐indifferent	41–119
*Streptanthus breweri*	BRE	Serpentine	Serpentine	51–241
*Streptanthus hesperidis*	HES	Serpentine	Serpentine	81–119
*Streptanthus polygaloides*	POL	Serpentine	Serpentine	48–175
*Streptanthus tortuosus*	TORW	Serpentine	Serpentine‐indifferent, primarily nonserpentine	53–424
*Caulanthus crassicaulis*	CRA	Nonserpentine	Nonserpentine	20–43
*Streptanthus diversifolius*	DIV	Nonserpentine	Nonserpentine	71–124
*Streptanthus farnsworthianus*	FAR	Nonserpentine	Nonserpentine	23–41

*Note*: Rainfall data obtained from The Calflora Database. Serpentine affinity describes the likelihood of finding a particular plant on serpentine soil, from serpentine‐indifferent to endemic. Serpentine‐indifferent plants are equally found on serpentine and non‐serpentine soils and serpentine endemic species are only found on serpentine soils.

### 2.2 Study design and rhizoplane soil collection

Plants were a subset from a larger experiment as described previously (Pearse et al., 2020). In summary, seeds from each plant species were collected from natural populations then germinated in a planting tray on a mist bench at the UC Davis Orchard Park greenhouse facility starting in November 2015. From December 2015 to January 2016, seedlings at the cotyledon stage were transplanted into their native soil in containers (Stuewe & Sons, St. Louis, MO, USA) in an outdoor lath house at ambient temperature at the Orchard Park facility. The native soils were collected along with the seeds from field populations. The experiment imposed seven watering treatments ranging between 8 and 238 mm/month and a subset of these treatments were used for this study. These levels were chosen to represent a field‐relevant range of growing season precipitation based on 30 years of precipitation records experienced by this group of species at our collection sites. Plants were placed in a completely randomized design across the greenhouse.

In June of 2016, rhizoplane soil was collected from flowering plants. A total of 72 samples (3 reps × 3 watering treatments × 8 plant species) were used for this experiment and they included three replicates of eight plant species from three watering treatments, low (16 mm/month = ~13 mL/week), mid (105 mm/month = ~87 mL/week), and high (194 mm/month = ~161 mL/week) (Pearse et al., 2017, 2020).

Rhizoplane soil was collected from roots of individual plants by first shaking the entire root system in a 0.9% (w/v) NaCl solution in a 50‐mL centrifuge tube. The roots were transferred into another 50‐mL centrifuge tube fill with 20 mL of 0.1% (v/v) Tween80 in 0.9%NaCl solution (Barillot et al., 2013). The tube and solution were shaken on a lateral shaker at 10 spm for 90 min. After shaking, the roots were removed and the tubes containing rhizoplane soil were centrifuged at 200 *g* for 10 min. The supernatant was discarded, and the pellet used to extract DNA using ZR Soil Microbe DNA MicroPrep following manufacturer's instructions (Zymo Research, Irvine, CA).

### 2.3 Library preparation and sequencing

From DNA extracts, the V4 region of the 16S SSU rRNA was amplified using primers 515F‐806R (515F: 5′ ‐ GTGCCAGCMGCCGCGGTAA ‐ 3′; 806R: 5′ ‐ GGACTACHVGGGTWTCTAAT ‐ 3′) (Caporaso et al., 2010). PCR was carried out in 25 μL reactions including 1 μL genomic DNA, 0.5 μL of each 10 μM primer, 12.5 μL of MyTaq Hot Start Red Mix (Bioline), and 10.5 μL of dH_2_O. PCR reactions were set up on ice to minimize non‐specific amplification and primer dimerization. PCR conditions were: denaturation at 94°C for 2 min; 34 amplification cycles of 30 s at 94°C, 30 s at 51°C, and 30 s at 72°C; followed by a 10 s final extension at 72°C. PCR products were visualized using gel electrophoresis and successful samples cleaned using Carboxyl‐modified Sera‐Mag Magnetic Speed‐beads in a PEG/NaCl buffer (Rohland & Reich, 2012).

Cleaned PCR products were quantified using the Qubit HS‐DS‐DNA kit (Invitrogen, Carlsbad CA), pooled in equimolar concentration and samples were submitted to the Centre for Comparative Genomics and Evolutionary Bioinformatics Integrated Microbiome Resource at Dalhousie University for sequencing using the Illumina MiSeq platform (500 cycles v2 PE250).

### 2.4 Bioinformatics

Amplicon sequence variants (ASVs) from 16S rRNA amplicons were identified using DADA2 (v1.7.2) (Callahan, McMurdie, et al., 2016). Briefly, paired‐end fastq files were processed by filtering and truncating forward reads at position 280 and reverse reads at position 200. Sequences were dereplicated, merged, and error‐corrected. Chimeras were removed, and the taxonomy assigned using the SILVA database (v128) (Glöckner et al., 2017; Quast et al., 2012; Yilmaz et al., 2014). A phylogenetic tree based on 16S sequences was estimated using the DECIPHER package (v2.8.1) in R to perform multi‐step alignment and phangorn (v2.4.0) to construct the 16S tree (Schliep, 2011; Wright et al., 2006). The sequence table, taxonomy, and metadata were combined into a phyloseq object and used for further analysis (phyloseq v1.30.0) (Callahan, Sankaran, et al., 2016; McMurdie & Holmes, 2013). Potential contaminants were identified using the frequency method from the decontam R package. Sequences annotated as mitochondria and chloroplast were then removed and the data were normalized.

### 2.5 Statistical analysis

To visualize the community composition of the microbial communities associated with each plant species across watering treatments, the relative abundance of taxa was visualized by aggregating taxa to the phylum level and filtering out taxa below 2%.

Shannon diversity accounts for both evenness and abundance as a measure of diversity and was used as the dependent variable in a linear regression model to determine the impact of soil affinity, watering treatment, and plant species on alpha diversity. Two models were conducted to test the interaction between affinity and watering treatment as well as plant species and watering treatment. First, species and watering treatment were used as independent variables and affinity as the random effect. Then, affinity and watering treatment were used as independent variables and species were used as the random effect. When applicable, Tukey's HSD post hoc tests were performed to determine which treatments differed.

We examined if bacterial 16S composition varied with plant soil affinity, watering treatment, or plant species using Bray–Curtis dissimilarity as a dependent variable in Permutational Multivariate Analysis of Variance (PERMANOVA), with affinity and watering treatment as the independent variables and species as a random effect. Similar to the test on diversity, a separate PERMANOVA was conducted with species and treatment as the independent variables and affinity as the random effect. To determine if watering treatment influenced relative abundance of sequences summed by bacterial genera, differential abundance analysis using DESeq2 (1.26.0) was used with watering treatment as a predictor. This approach is appropriate despite the compositional nature of the data due to its focus on relative abundances of the genera rather than absolute abundances. Additionally, random forest analysis was used to identify bacterial features of the rhizoplane associated with soil affinity or watering treatment. We separately examined homogeneity of variance among samples as a function of soil affinity, watering treatment, or species using betadisper.

To examine if plant species that were closely related were also similar in microbial community composition, we compared pairwise branch lengths among plant species (cophenetic phylogeny) and Bray–Curtis dissimilarity in 16S amplicon composition (all ASVs included) (Bray & Curtis, 1957; Lozupone & Knight, 2005). The correlation between these matrices were tested using a Mantel test and Pearson's correlation analysis. To explore if the natural watering regime influences the rhizoplane bacterial community of the species, general rainfall data was obtained from calflora.org and the centroids for each species under each experimental watering treatment were calculated using betadisper. The Euclidean distance of these centroids were regressed against the low rainfall using a linear model.

To examine if the results above were driven by dominant bacterial taxa, we separated ASVs into rare and dominant components based on specific cutoffs (Xu et al., 2021). MultiCola analysis was used to ensure the aforementioned cutoffs were appropriate (Gobet et al., 2010; Guo, 2022). The rare and dominant microbial components were analyzed using the same analyses as the full microbiome. Detailed description of these methods and results are outlined in the Data S1.

## 3 RESULTS

**FIGURE 1:**
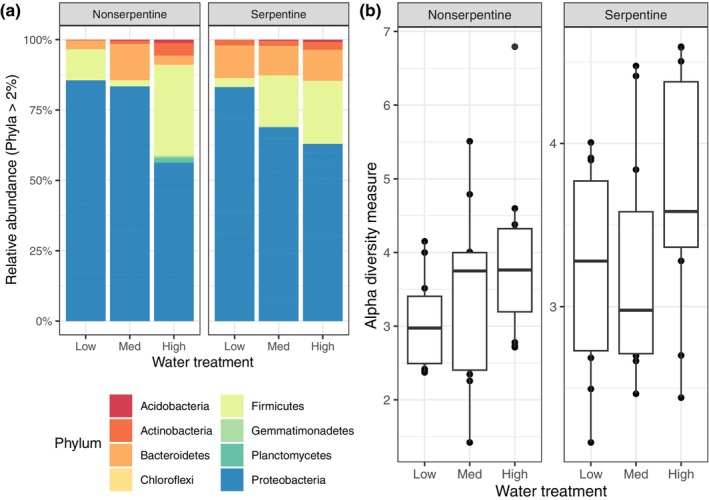
Relative abundance and alpha diversity of rhizoplane bacterial communities. (a) Proteobacteria dominated the communities across watering treatments and soil affinities. (b) The richness of the rhizoplane bacterial communities decreased significantly in low watering treatments compared to the high watering treatment (*F*_2,60_ = 3.759, *p* = .029). The horizontal line within the box represents the median values while the vertical lines represent the minimum and maximum data values. The single dot not on a line represents an outlier data point.

After quality filtering and removal of non‐target sequences, we recovered 3,603,995 reads (average 50,005 per sample) that were grouped into 10,554 amplicon sequence variants. Sampling curves within samples were saturating, indicating a robust sampling of the microbial diversity associated with individual plants. Most samples were predominated by *Proteobacteria* and in plants receiving the most water, *Firmicutes* increased in relative abundance (Figure 1a and Figure S1).

### 3.1 How does watering treatment and soil affinity impact alpha diversity of bacteria in the rhizoplane?

In both models, greater water availability increased Shannon diversity of bacterial communities in the rhizoplane of *Streptanthus* (Figure 1b; Soil affinity as independent variable: *F*_2,60_ = 3.759, *p* = .029; Plant species as independent variable: *F*_2,48_ = 5.354, *p* = .008). Bacterial alpha diversity was similar across plant species (Figures S1 and S2; *F*_7,48_ = 1.017, *p* = .431) and soil affinity (*F*_1,60_ = 0.124, *p* = .726), but plant species varied in their response to watering treatment (species × watering – *F*_14,48_ = 2.754, *p* = .005). Soil affinity did not predict response to watering treatment (affinity × watering – *F*_2,60_ = 0.389, *p* = .679).

### 3.2 How does watering treatment shift community composition of rhizoplane bacteria and which bacteria are enriched under drought?

**FIGURE 2:**
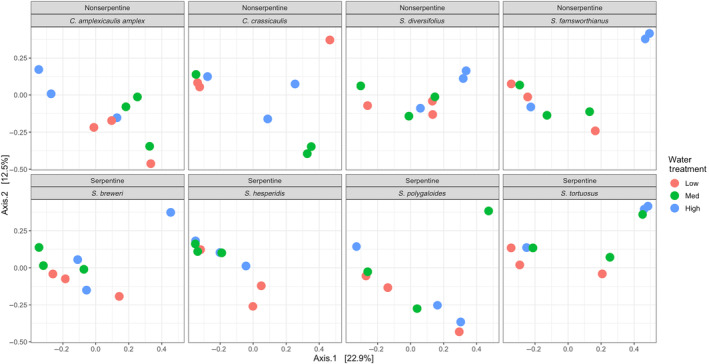
NMDS of rhizoplane samples across watering treatment using Bray–Curtis dissimilarity. Points that are closer together have smaller dissimilarity values meaning they are more similar to each other. Rhizoplane microbial communities become more similar as water stress increases (PERMANOVA: *F*_2,71_ = 1.81, *p* = .014, *R* ^2^ = .047; betadisper: *F*_2,69_ = 3.78, *p* = .033). Points represent individual plants sampled.

Bacterial species composition differed with watering treatment (Figure 2; *F*_2,71_ = 1.81, *p* = .014, *R* ^2^ = .047), soil affinity (*F*_1,71_ = 1.85, *p* = .041, *R* ^2^ = .024), and among plant species (*F*_6,71_ = 1.64, *p* = .004, *R* ^2^ = .127) and there was no significant interaction between watering treatment and soil affinity (*F*_2,71_ = 1.04, *p* = .358, *R* ^2^ = .027) or watering treatment and plant species (Plant species as main effect: *F*_14,71_ = 0.999, *p* = .448, *R* ^2^ = .181). In addition, low watering decreased the variance among samples in microbial composition (*F*_2,69_ = 3.78, *p* = .033), but homogeneity of variance among samples was not significantly predicted by plant species (*F*_7,64_ = 1.72, *p* = .124) or soil affinity (*F*_1,70_ = 2.06, *p* = .172).

**FIGURE 3:**
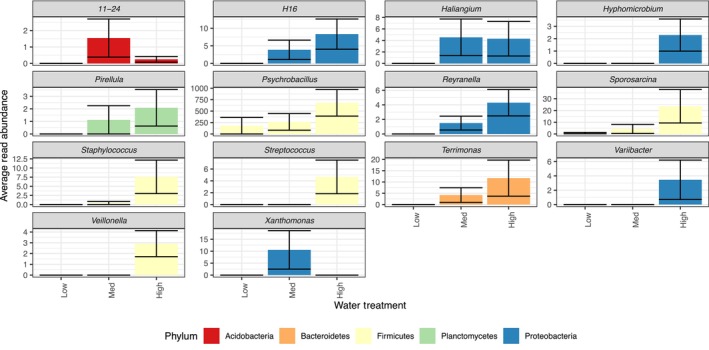
Differential abundances of genera between water treatments using DESeq2. Average read abundance of genera that were shown to be differentially abundant between watering treatments in the rhizoplane at alpha = 0.05. All genera differ significantly between treatments. Bars represent average read abundance with standard error across all plant species.

Differential abundance analysis using DESeq2 showed that several genera were impacted by watering treatment (Figure 3). The differences in relative abundances were largely driven by the absence of some genera in the low and medium watering treatment. Some phyla had only one genus that significantly changed in relative abundance across watering treatment, whereas others, like Proteobacteria and Firmicutes, had multiple genera impacted by the treatment. Of the Proteobacteria, *H16*, *Reyranella*, and *Variibacter* increased in relative abundance in the high watering treatment and were absent in the low watering treatment. *Haliangium*, *Hyphomicrobium*, and *Xanthomonas* were also absent from the low watering treatment, but present in either mid or high watering treatments. Firmicutes including *Psychrobacillus*, *Sporosarcina*, and *Staphylococcus*, Bacteroidetes such as *Terrimonas* as well as Planctomycetes like *Pirellula* were most abundant in the high watering treatment.

**FIGURE 4:**
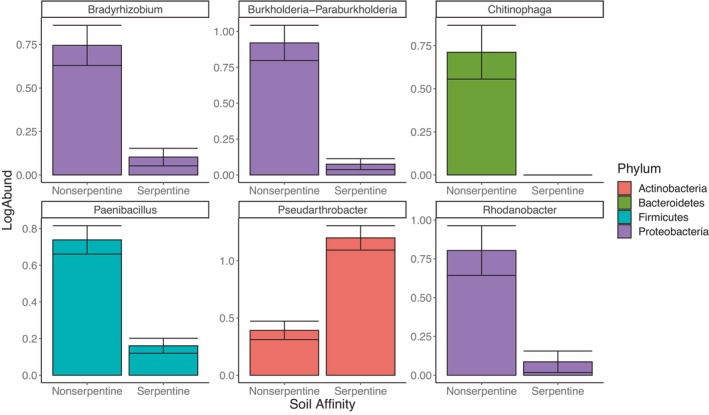
Bar plot with log transformed abundances of taxa that were identified as features of serpentine and nonserpentine soils using random Forest analysis. The out‐of‐box error for this analyze was 9.72%.

Random forest analysis identified bacterial features associated with affinity to serpentine and nonserpentine soils with an out‐of‐box error rate of 9.72%. Examples of some of these taxonomical features include *Bradyrhizobium*, *Chitinophaga*, *Rhodanobacter*, and *Paenibacillus* which are associated with affinity to nonserpentine soils and *Pseudarthrobacter* that is associated with affinity to serpentine soils (Figure 4). The out‐of‐box error for random forest analysis of watering treatments was 44.44%, therefore we did not examine taxa that differentiate treatments.

### 3.3 Does plant phylogeny or natural water regime influence the response of rhizoplane microbial communities to water stress?

Among the plant species examined here, rhizoplane bacterial community composition (Bray‐Curtis distance of OTUs) was not significantly associated with phylogenetic distance among plant species (*r* = .037; *p* = .153). Furthermore, the natural water regime was not significantly correlated with the observed variation of the rhizoplane bacterial communities of the plants (*F*_1,70_ = 0.33, *p* = .568).

### 3.4 Do results differ if all bacterial taxa or only rare members are considered?

When the rare members of the bacterial community are considered: the alpha diversity was lower in the low watering treatment of plants with an affinity to nonserpentine soils (Figure S3; *p* = .04). Affinity to serpentine soil did not influence the bacterial alpha diversity in the different watering treatments (soil affinity: *p* = .10). Additionally, phylogeny was significantly correlated with bacterial community similarity in the rare microbiome (Figure S4; *r* = .1579, *p* = <.0001).

## 4 DISCUSSION

The Streptanthoid Complex is a group of at least 53 species of *Streptanthus* and *Caulanthus* with a distribution across California and varying affinities to serpentine soils (Cacho et al., 2014). Serpentine soils are soils with naturally high amounts of heavy metals and low plant nutrients. Using the *Streptanthus* genus and species that span a gradient of naturally stressful soil, we tested multiple hypotheses starting with (1) affinity to serpentine soil correlated with greater bacterial alpha diversity under simulated drought compared to plants with an affinity to nonserpentine soils, then (2) rhizoplane bacterial communities from species with an affinity to serpentine soils will be more similar to each other than to species with an affinity to nonserpentine soils, and finally (3) that plant phylogeny influences the response of rhizoplane bacterial communities under water stress.

### 4.1 Alpha diversity decreases and rhizoplane bacterial communities become more similar as water levels decrease

Alpha diversity is a measure of the observed species in a sample and one way to define biodiversity. There is a positive correlation between biodiversity and ecosystem functioning; therefore, understanding how stress impacts the alpha diversity of plant‐associated bacteria can provide some information about how ecosystem functions can be affected (Jochum et al., 2020). A legacy effect is a phenomenon by which microbial communities that are pre‐exposed to a stress and better able to withstand subsequent stresses of the same nature (Subedi et al., 2022). We predicted that plants and microbes associated with serpentine soils would not be as impacted by water stress as those with an affinity to nonserpentine soils since serpentine soils can have low water‐holding capacity. Although we hypothesized that plant soil affinity would predict the effect of watering on the diversity of bacterial communities, our analyses did not support this hypothesis. Additionally, our results show that simulated drought shifts rhizoplane microbial communities to be more similar as water levels decrease.

The conserved shift in rhizoplane bacterial communities observed in this study is somewhat remarkable given that each species was grown in its own, field‐collected soil; soils were of different parent material, and many collection sites were hundreds of miles apart. Still, the relative abundance of several bacterial genera spanning five phyla differed significantly across watering treatments. Genera in the Firmicutes including *Psychrobacillus*, *Streptococcus*, *Veillonella*, *Sporosarcina*, and *Staphylococcus* were more abundant in the highest watering treatment. *Psychrobacillus* is a nitrogen‐fixing bacterium often found as a plant endophyte (Alishahi et al., 2020; Rilling et al., 2018) and has been shown, along with *Sporosarcina*, to have plant growth‐promoting properties like phosphorus solubilization and the production of phytohormones (Verma et al., 2015; Xu et al., 2018). In this study, the Bacteroidetes, *Terrimonas*, were abundant in the high‐watering treatment and they have been shown to be enriched after drought (Acosta‐Martínez et al., 2014; Meisner et al., 2018). *Pirellula*, a member of the Planctomycetes, were also the most abundant in the highest watering treatment (Figure 3) and they have been implicated as a plant growth‐promoting bacteria in lettuce (Cipriano et al., 2016). In the low‐watering treatment, these bacterial groups almost disappear from the microbial community. Identifying, isolating, and characterizing drought‐adapted microorganisms will be necessary if microbes will be used to assist plant adaptation to climate change.

Simulated drought significantly decreased the bacterial diversity and evenness in the rhizoplane across most *Streptanthus* species sampled here regardless of affinity to serpentine soil. Generally, Gram‐positive bacteria are resistant to drought due to their thick peptidoglycan cell wall (Naylor et al., 2017) so it is expected that Gram‐positive bacteria would increase under water stress. Interestingly, at the phylum level, Gram‐positive Firmicutes decreased and Gram‐negative Proteobacteria were enriched under low‐water conditions (Fuchslueger et al., 2014; Schimel et al., 2007). This divergence from the expected could be due to the initial differences in the bacterial community of field soils. However, it is worth exploring what mechanisms underlie the reduction of Gram‐positive bacteria under water stress.

Changes in bacterial diversity and relative abundances likely contributed to the observed rhizoplane bacterial community similarity. Previous research found similar results with C3 and C4 grasses with varying tolerance to water stress (Naylor et al., 2017) and conserved shifts in microbial communities have previously been demonstrated (Fitzpatrick et al., 2018; Xu & Coleman‐Derr, 2019). This study shows conserved shifts in the rhizoplane and future research can examine the endosphere to identify if microbial communities react similarly across rhizosphere compartments. Some studies document a reduction in bacterial diversity under water stress (Fahey et al., 2020; Graham et al., 2016; Lau & Lennon, 2011; Preece et al., 2019; Prudent et al., 2020) while others find little to no effect (Bachar et al., 2010; Tóth et al., 2017). Due to the divergent trends in the effect on watering on alpha diversity, this result in *Streptanthus* adds dimensionality. Still, a larger sample size may offer some more nuance into how rhizoplane bacterial communities associated with serpentine soils respond to stress including stresses not tested in this study.

### 4.2 Bacterial features of plant affinity to serpentine and nonserpentine soils

Bacteria in the rhizosphere are often recruited from surrounding soil by plant root exudation and closely related plants may have similar exudation profiles. Bacterial abundance is often increased, and diversity is reduced in the rhizosphere compared to surrounding soil and these microbes generally support plant health (Ling et al., 2022). Therefore, understanding how bacteria in the rhizoplane of these closely related species respond to stress can be useful for conserving *Streptanthus*. Several bacteria were identified as taxonomic features representing affinity to serpentine (*Pseudarthrobacter*) or nonserpentine (*Bradyrhizobium*, *Chitinophaga*, *Rhodanobacter* and *Paenibacillus*) soils. Associating with bacteria can enhance plant fitness in various soil conditions. For example, *Pseudarthrobacter* produces indole‐3‐acetic acid (IAA), a phytohormone that promotes root growth and is recruited by tall fescue to tolerate salt stress (Li et al., 2022). It has been found in post‐mining sites and supports the growth of legumes on contaminated heavy‐metal sites (Oubohssaine et al., 2022). Serpentine soils have high amounts of heavy metals and magnesium salt stress; therefore, members of *Streptanthus* with an affinity to serpentine soil associating with this genus is logical. *Bradyrhizobium* are known for their ability to fix nitrogen (Ormeño‐Orrillo & Martínez‐Romero, 2019) and *Chitinophaga* degrade biomass, primarily chitin (Glavina Del Rio et al., 2010). Nitrogen fixation is a valuable plant growth‐promoting property that allows plants to access atmospheric nitrogen and the ability to degrade the primary component of fungal cell walls contributes to the global carbon cycle. *Rhodanobacter* are commonly denitrifying and convert nitrates in the soil into atmospheric nitrogen thereby supporting the nitrogen cycle (Green et al., 2012). *Paenibacillus* have generally been studied for their multiple plant growth‐promoting properties (Jeong et al., 2019). Nonserpentine soils generally have substantial plant litter and increased competition due to their high plant productivity; bacterial communities that can thrive in those conditions are crucial for nonserpentine ecosystem. Conservation practices that are designed for the protection of endemic plants and serpentine soils should also consider isolating and preserving these microbes to support the growth and development of the plants and ecosystem services of the soil.

### 4.3 Closely related *Streptanthus* species do not host more similar rhizoplane bacterial communities

The similarity of the rhizoplane bacterial communities was influenced by watering treatment, but not soil affinity. The bacterial rhizoplane community was not more similar in more closely related species. Members of the same genus often have similar rhizosphere communities (Burns et al., 2015) and rare taxa are often recruited into the rhizosphere (Saleem et al., 2016). This work shows that members of the *Streptanthus* genus also harbor a similar rhizoplane community that is not distinct between species. However, the rare microbiome is distinct between species. This result is intriguing considering these species were collected from diverse geographic areas and grown on their home soils. Even with varying indigenous bacterial communities, *Streptanthus* species may select for similar rhizoplane communities. It will be helpful for future research to explore the processes that impact taxonomical and functional rhizosphere microbial recruitment and how they will respond to global climate change. This information will allow us to develop conservation practices that can mitigate negative impacts and preserve rare and endemic plant species like members of the *Streptanthus* genus. It may be worth exploring the genetic basis of the composition of bacterial rhizosphere communities in *Streptanthus*. Additionally, measuring the quality and quantity of root exudates of species within the *Streptanthus* spp. can help us better understand what influences the rhizoplane microbial communities.

## 5 CONCLUSION

It is important to understand the mechanisms of plant–microbe associations if we are to preserve biodiversity in the face of increasingly variable water regimes, yet these interactions can be context dependent and conditioned by the host plant species (Ishaq et al., 2017; Isobe et al., 2020; Jorquera et al., 2017). Because serpentine soils are naturally drought‐prone, we hypothesized that the rhizoplane microbial communities associated with plants with a serpentine‐affinity would be less impacted by drought due to legacy effects. However, we did not find support for that hypothesis. Most species in the *Streptanthus* clade grow in well drained, coarse soils (Cacho & Strauss, 2014). This may mean that organisms on serpentine soils are not uniquely situated to resist unusual climatic events or that *Streptanthus* species are generally adapted to drought, whether they are associated with serpentine or not.

The rhizoplane bacterial community shifted under water stress to be more similar in all watering treatments. Still, microbes from drier areas have been shown to use carbon more efficiently than microbes from wetter areas even as their compositions remain the similar under water stress (Leizeaga et al., 2021). Future research would be useful to discern if microbes from serpentine soils use resources more effectively than microbes from nonserpentine soils.

Although our experimental conditions only approximated drought, the inflection point of fitness (i.e., the amount of water at which adding more water most increased plant fitness) of most of these populations occurred at the watering level reflecting mean annual precipitation for that field collection site (averaged over the last 30 years), suggesting watering levels used here are biologically relevant (Pearse et al., 2020). This is important because there a different types of droughts which can be characterized based on a lack of precipitation over different time spans as well as increased temperatures and other ecological changes (Hoover & Rogers, 2016). Future studies under actual field drought conditions would be useful, particularly if drought duration and watering frequency are important in determining bacterial community responses.

## AUTHOR CONTRIBUTIONS

**Alexandria Igwe:** Conceptualization (equal); data curation (equal); formal analysis (lead); investigation (lead); methodology (lead); project administration (lead); validation (lead); visualization (lead); writing – original draft (lead); writing – review and editing (lead). **Rachel L. Vannette:** Conceptualization (equal); formal analysis (supporting); methodology (equal); resources (lead); supervision (lead); writing – original draft (supporting); writing – review and editing (equal). **Ian S. Pearse:** Conceptualization (equal); data curation (lead); writing – review and editing (supporting). **Jessica M. Aguilar:** Data curation (lead); writing – review and editing (supporting). **Sharon Y. Strauss:** Conceptualization (equal); data curation (lead); funding acquisition (lead); writing – review and editing (equal).

## CONFLICT OF INTEREST STATEMENT

Authors declare no conflict of interest.

## Supporting information


Data S1.

Figures S1–S4.

## Data Availability

Raw sequences are available at www.ncbi.nlm.nih.gov/sra/PRJNA613384. Data and code available at https://github.com/anigwe/streptanthus_drought.
