# Supplementary material for: Plant species within Streptanthoid Complex associate with distinct microbial communities that shift to be more similar under drought
Source: Ecol Evol. 2024 Mar 24;14(3):e11174. doi: 10.1002/ece3.11174 (PMC10961476; doi:10.1002/ece3.11174)
Supplement: Supplementary file 1 — Data S1. [file ECE3-14-e11174-s001.docx]

SUPPLEMENTARY METHODS

Taxa were divided into rare and dominant taxa using unnormalized data. Taxa were categorized operational taxonomic units (OTUs) based on their relative abundance in a set of samples. Six categories were defined: always abundant taxa (AAT) were those that had a relative abundance of at least 1% in all samples; conditionally abundant taxa (CAT) were those that had a relative abundance of at least 1% in some samples, but never less than 0.01%; always rare taxa (ART) were those that had a relative abundance of less than 0.01% in all samples; conditionally rare taxa (CRT) were those that had a relative abundance of less than 1% in all samples, but less than 0.01% in some samples; moderate taxa (MT) were those that had a relative abundance between 0.01% and 1% in all samples; and conditionally rare and abundant taxa (CRAT) were those that had a relative abundance ranging from rare (less than 0.01%) to abundant (at least 1%). For further analyses, the AAT, CAT, and CRAT were combined as abundant taxa, while the ART and CAT were combined as rare taxa (Xu et al. 2021). The “group_taxa” function from the “ecoloop” package was used for MultiCola analysis to ensure the aforementioned cutoffs were appropriate (Gobet et al. 2010; Guo 2022).

##

## SUPPLEMENTARY RESULTS

After quality filtering and removal of non-target sequences and contaminants, we recovered 3,603,995 reads (average 50,005 per sample) that were grouped into 10,477 amplicon sequence variants (ASVs). There were 302 ASVs that were considered “Dominant Taxa” and 10,175 ASVs that were considered “Rare Taxa.” Sampling curves within samples were saturating, indicating a robust sampling of the microbial diversity associated with individual plants.

The relative abundance of the dominant and all taxa were not impacted by watering treatment, soil affinity, or species identity (Supplementary Figure 3A). Simulated drought did impact the relative abundance of the rare taxa (F_2,1356_ = 5.232, *P* = 0.005) and the Shannon diversity of the full (Shannon: F_2,60_ = 3.759, *P* = 0.029) and rare (Shannon: F_2,60_ = 7.993, *P* = <0.001) bacterial communities in the rhizoplane of *Streptanthus* species (Supplementary Figure 3B). Post-hoc test showed that, for the rare microbiome, the alpha diversity was lower in the low watering treatment compared to the high watering treatment of plants with an affinity to nonserpentine soils (*P* = 0.04). Alpha diversity was not significantly different based on watering treatment in the rhizoplane of plants with an affinity to serpentine soils (*P* = 0.10).

Soil affinity had a significant influence on the similarity between rare and combined bacterial community, but not the dominant taxa (Supplementary Table 1). Watering treatment and species identity both significantly influenced the rare, dominant, and combined bacterial communities. The interaction between soil affinity and watering treatment significantly impacted the rare microbiome. In all community types (rare, dominant, and all), soil affinity was responsible for structuring 2% of the bacterial community, watering treatment was responsible for ~5% of the variation of the bacterial community, and species identity was responsible for ~12% of the variation in the bacterial community. The interaction between soil affinity and watering treatment is responsible for structuring about 3% of the bacterial rhizoplane community.

Phylogeny was significantly correlated with bacterial community similarity in the rare microbiome (Supplementary Figure 4; *r*=0.1579, *P*=<0.0001), but not the dominant (*r*=0.0084, *P*=0.369), and combined microbiome (*r*=0.0365, *P*=0.1525).

Supplementary Table 1 – Analysis of variance was used to identify the effect of predictors on bacterial community simililarity as measured using the Bray-Curtis metric. The community was measured using 16S rRNA amplicon sequencing obtained from rhizoplane soil from members of the *Streptanthus* clade (Plant Species). Members of this clade have varying affinity to serpentine and nonserpentine soils (Soil Affinity) and, in the experiment, treated with a low, mid, or high watering regime (Water Treatment).

|  |  |  | Rare Taxa | | | Dominant Taxa | | | All Taxa | | |
| --- | --- | --- | --- | --- | --- | --- | --- | --- | --- | --- | --- |
| Predictor | df | n | F | R^2^ | *P* | F | R^2^ | *P* | F | R^2^ | *P* |
| Soil Affinity | 1 | 72 | 2.03 | 0.03 | 0.001 | 1.70 | 0.02 | 0.072 | 1.85 | 0.02 | 0.041 |
| Water Treatment | 2 | 72 | 1.55 | 0.04 | 0.001 | 1.81 | 0.05 | 0.012 | 1.81 | 0.05 | 0.014 |
| Plant Species | 6 | 72 | 1.60 | 0.12 | 0.001 | 1.57 | 0.12 | 0.010 | 1.64 | 0.13 | 0.004 |
| Soil Affinity*Water Treatment | 2 | 72 | 1.15 | 0.03 | 0.038 | 0.91 | 0.02 | 0.583 | 1.04 | 0.03 | 0.358 |
