# Supplementary material for: Plant species within Streptanthoid Complex associate with distinct microbial communities that shift to be more similar under drought
Source: Ecol Evol. 2024 Mar 24;14(3):e11174. doi: 10.1002/ece3.11174 (PMC10961476; doi:10.1002/ece3.11174)
Supplement: Supplementary file 2 — Figures S1–S4. [file ECE3-14-e11174-s002.zip › Supplementary Figure Captions.docx]

Supplementary Figure Captions

Supplementary Figure 1 - Stacked bar plot of the relative abundance of phyla from the rhizoplane of various members of the Streptanthoid Complex. *Proteobacteria* predominates in most samples, especially in the low watering treatment. *Bacteroidetes* and *Firmicutes* are also major components of the rhizoplane community.

Supplementary Figure 2 - Whisker plot of Shannon diversity index of the rhizoplane microbial community associated with various members of the Streptanthoid Complex. Each plot shows the range and the median of the alpha diversity of three replicates of each species and watering treatment pair.

Supplementary Figure 3 - Stacked bar plots (A) and whisker plot (B) of the relative abundances of phlya from the rare, dominant, and combined rhizoplane of various members of the Streptanthoid Complex aggregated by watering treatment (24 replicates). The whisker plot shows the median (black line within rectangle) and range of alpha diversity.

Supplementary Figure 4 - Principal coordinate analysis (PCoA) of the rare, dominant, and combined rhizoplane community of various members of the Streptanthoid Complex. Points are colored by watering treatment and represent one replicate. They are organized from top to bottom in accordance with their relatedness which is represented by the phylogenetic tree.
