# Supplementary material for: Plant species within Streptanthoid Complex associate with distinct microbial communities that shift to be more similar under drought
Source: Ecol Evol. 2024 Mar 24;14(3):e11174. doi: 10.1002/ece3.11174 (PMC10961476; doi:10.1002/ece3.11174)
Supplement: Supplementary file 2 — Figures S1–S4. [file ECE3-14-e11174-s002.zip › SupplementaryFigure3.pdf]

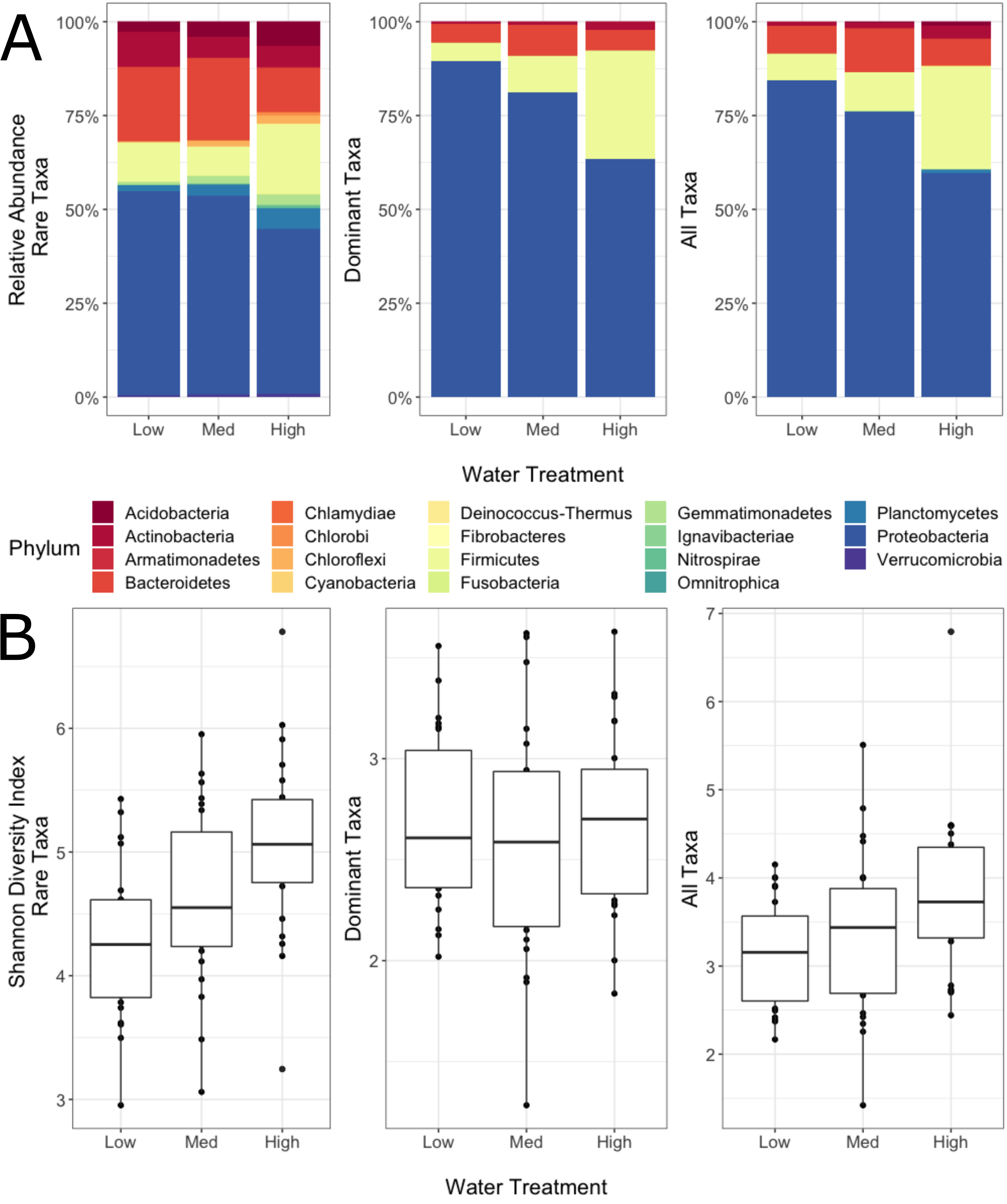

A
Relative Abundance
Rare Taxa
Dominant Taxa
All Taxa
100%
75%
50%
25%
0%
Low
Med
High
Water Treatment
Phylum
Acidobacteria
Actinobacteria
Armatimonadetes
Bacteroidetes
Chlamydiae
Chlorobi
Chloroflexi
Cyanobacteria
Deinococcus-Thermus
Fibrobacteres
Firmicutes
Fusobacteria
Gemmatimonadetes
Ignavibacteriae
Nitrospirae
Omnitrophica
Planctomycetes
Proteobacteria
Verrucomicrobia
B
Shannon Diversity Index
Rare Taxa
Dominant Taxa
All Taxa
Low
Med
High
Water Treatment
