# Supplementary figures and images for: Plant species within Streptanthoid Complex associate with distinct microbial communities that shift to be more similar under drought
Source: Ecol Evol. 2024 Mar 24;14(3):e11174. doi: 10.1002/ece3.11174 (PMC10961476; doi:10.1002/ece3.11174)

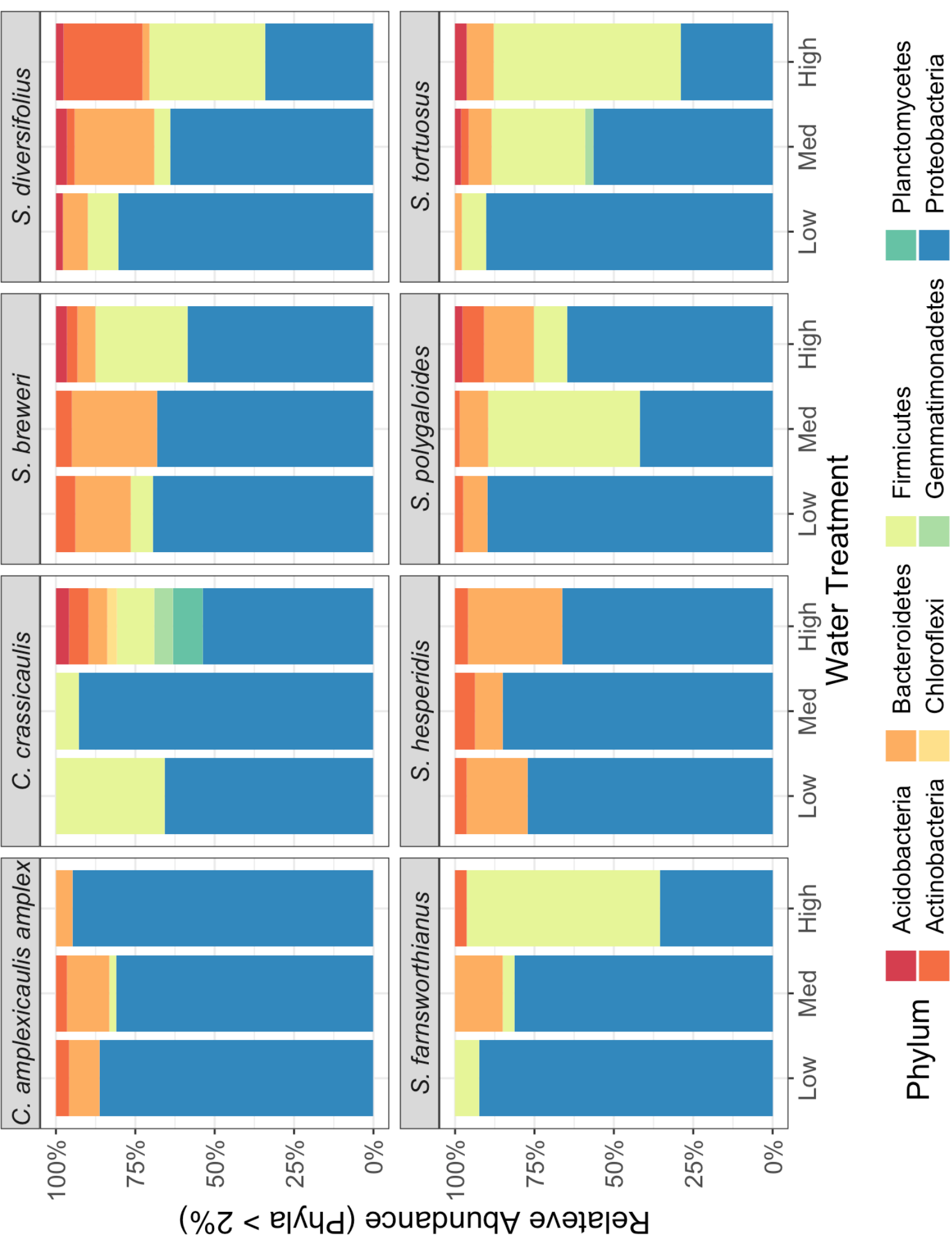

Supplement: Supplementary file 2 — Figures S1–S4. [file ECE3-14-e11174-s002.zip › supplementaryfigure1.pdf]

Alpha Diversity Indices

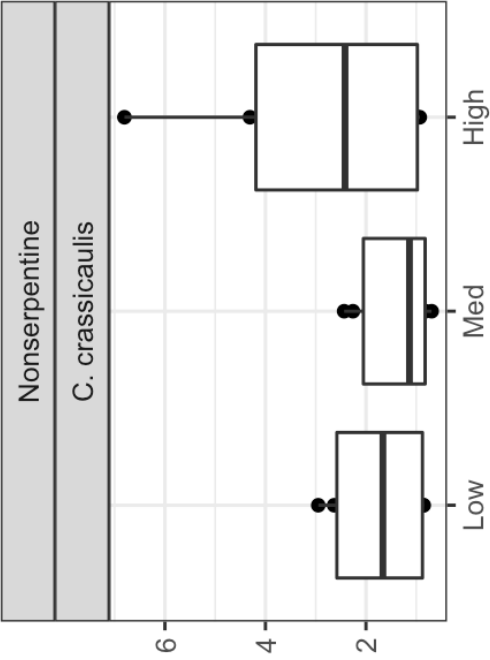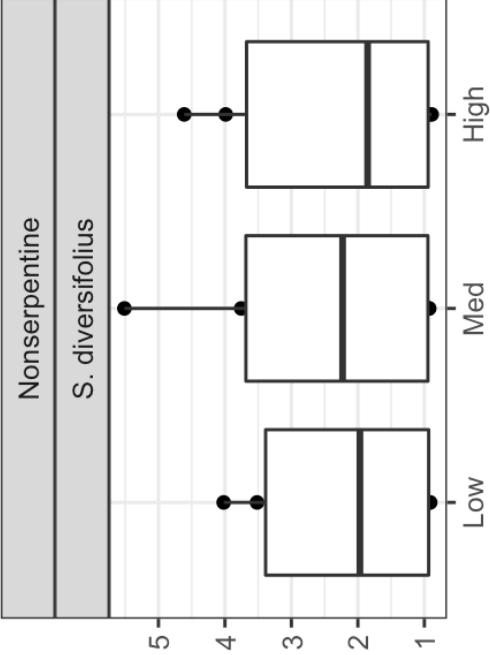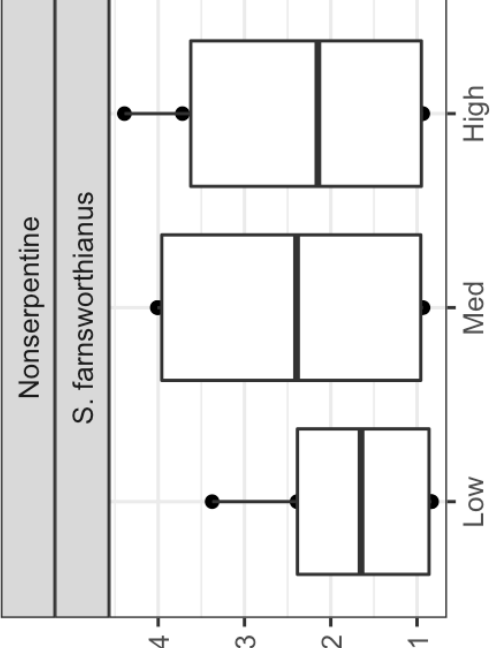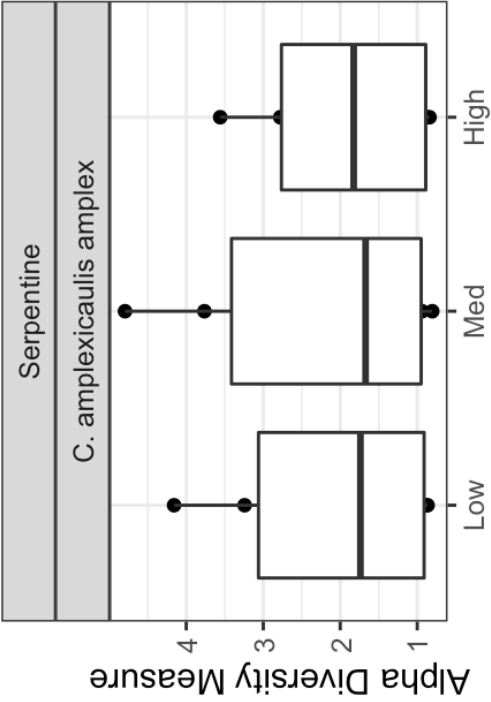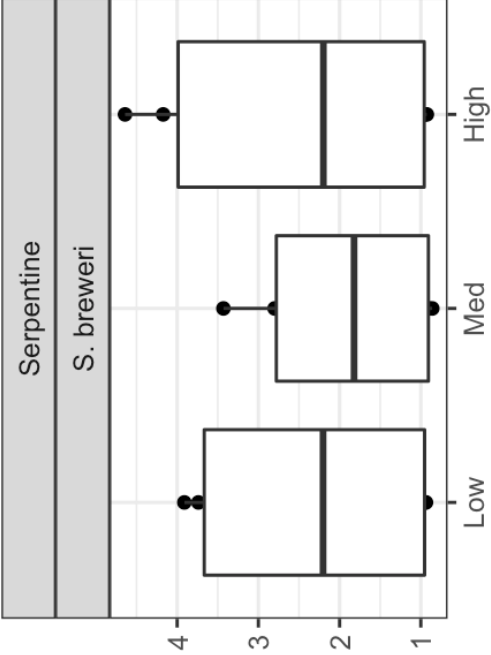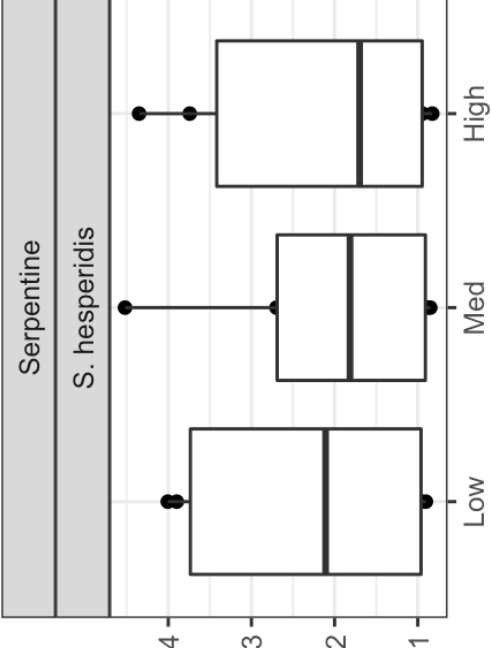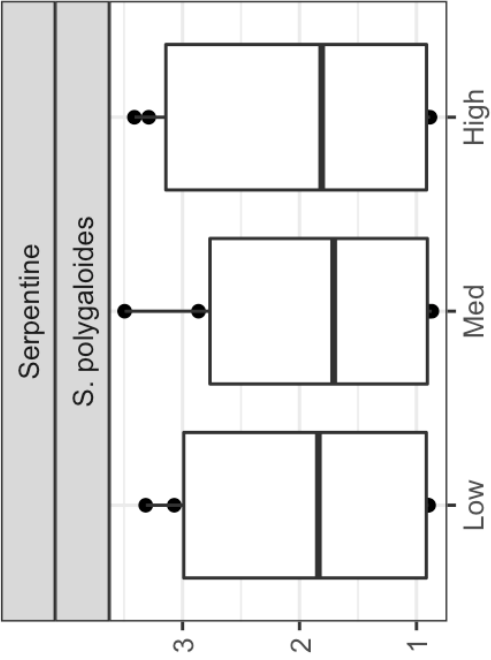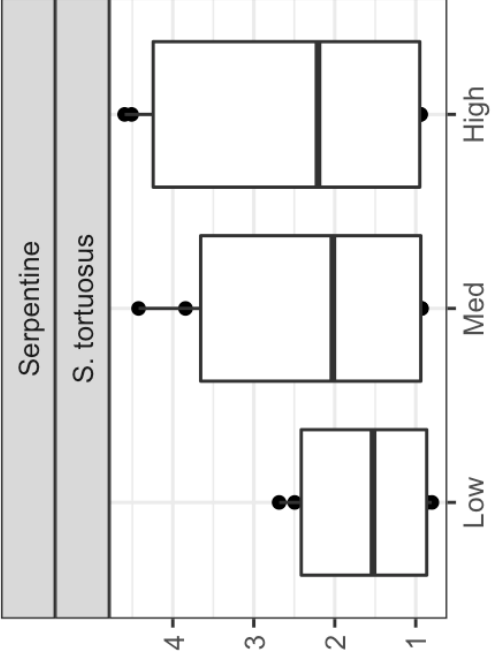

Water Treatment

Supplement: Supplementary file 2 — Figures S1–S4. [file ECE3-14-e11174-s002.zip › supplementaryfigure2.pdf]

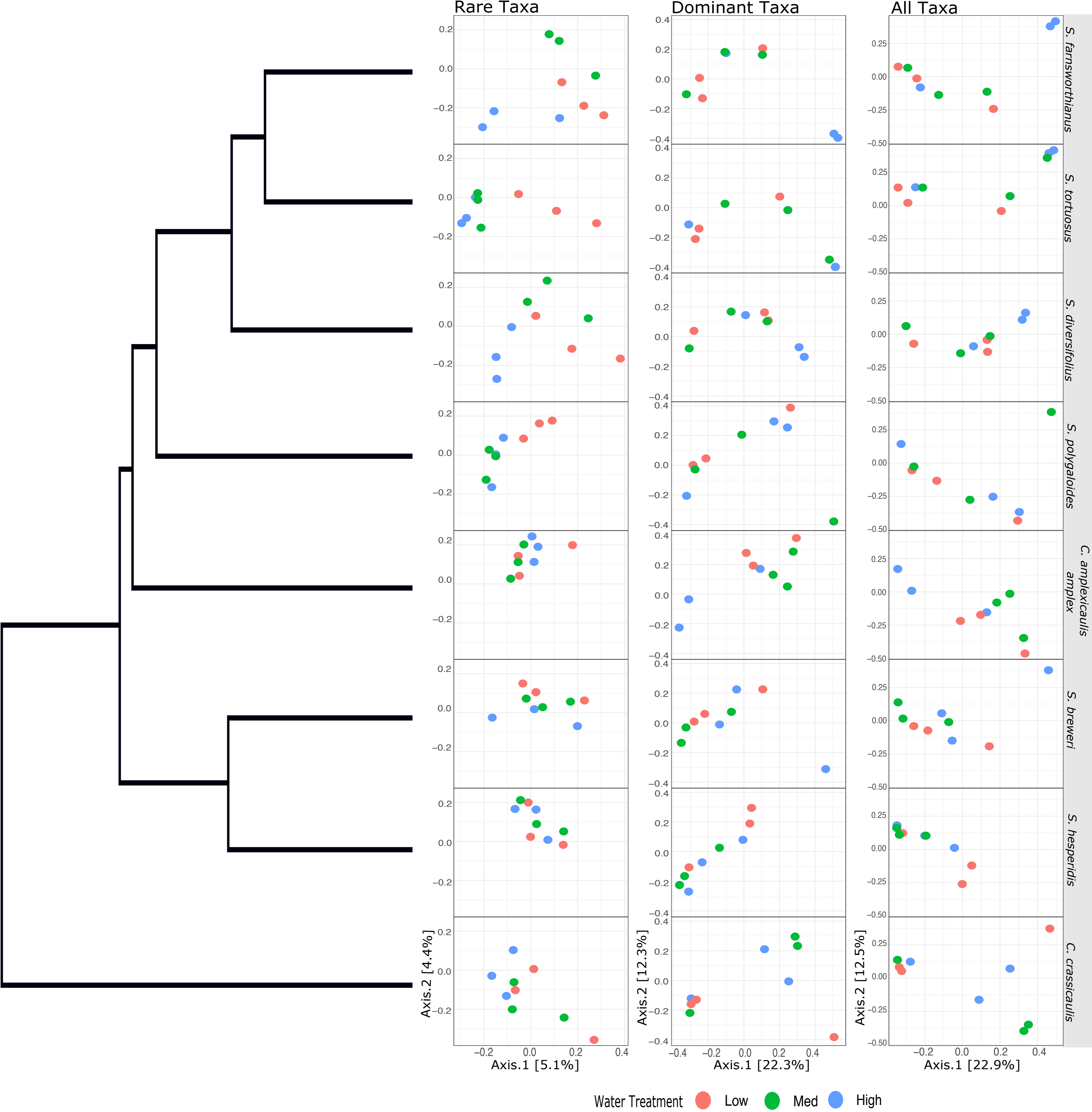

Supplement: Supplementary file 2 — Figures S1–S4. [file ECE3-14-e11174-s002.zip › SupplementaryFigure4.pdf]
